# Rumen and Fecal Microbial Community Structure of Holstein and Jersey Dairy Cows as Affected by Breed, Diet, and Residual Feed Intake

**DOI:** 10.3390/ani9080498

**Published:** 2019-07-29

**Authors:** Samantha J. Noel, Dana W. Olijhoek, Farran Mclean, Peter Løvendahl, Peter Lund, Ole Højberg

**Affiliations:** 1 Department of Animal Science, AU-Foulum, Aarhus University, DK-8830 Tjele, Denmark; 2 Department of Molecular Biology and Genetics, AU-Foulum, Aarhus University, DK-8830 Tjele, Denmark

**Keywords:** next-generation sequencing, microbiome, cattle breed, residual feed intake, methane

## Abstract

**Simple Summary:**

Dietary interventions aimed at reducing methane production may be influenced by other factors such as animal breed and feed efficiency (indicated by residual feed intake (RFI) status). We examined the rumen and fecal microbiota of Holstein and Jersey dairy cows with diverging RFI status fed diets differing in concentrate-to-forage ratio. Community differences seen in the rumen were reduced or absent in feces, except in the case of animal-to-animal variation, where differences were more pronounced. Understanding factors that influence methane production will be key to determining effective methane reduction strategies in the future.

**Abstract:**

Identifying factors that influence the composition of the microbial population in the digestive system of dairy cattle will be key in regulating these populations to reduce greenhouse gas emissions. In this study, we analyzed rumen and fecal samples from five high residual feed intake (RFI) Holstein cows, five low RFI Holstein cows, five high RFI Jersey cows and five low RFI Jersey cows, fed either a high-concentrate diet (expected to reduce methane emission) or a high-forage diet. Bacterial communities from both the rumen and feces were profiled using Illumina sequencing on the 16S rRNA gene. Rumen archaeal communities were profiled using Terminal-Restriction Fragment Length Polymorphism (T-RFLP) targeting the mcrA gene. The rumen methanogen community was influenced by breed but not by diet or RFI. The rumen bacterial community was influenced by breed and diet but not by RFI. The fecal bacterial community was influenced by individual animal variation and, to a lesser extent, by breed and diet but not by RFI. Only the bacterial community correlated with methane production. Community differences seen in the rumen were reduced or absent in feces, except in the case of animal-to-animal variation, where differences were more pronounced. The two cattle breeds had different levels of response to the dietary intervention; therefore, it may be appropriate to individually tailor methane reduction strategies to each cattle breed.

## 1. Introduction

The contribution of dairy cattle greenhouse gas emissions to climate change has prompted research into the function and structure of the rumen microbiome [1,2,3,4]. The rumen contains a complex community of microorganisms including archaea, bacteria, fungi and protozoa that ferment ingested feedstuffs, providing nutrients for the host and also the by-product methane. Compared to the rumen, the hindgut microbiota, represented by the fecal microbiome, is poorly characterized, particularly by next-generation sequencing. Next-generation sequencing allows for comprehensive surveys of microbiomes both quickly and inexpensively by targeting the 16S rRNA gene. The fecal microbiome has differences from the rumen microbiome [5,6] but, like the rumen community, the fecal community is altered by changes in diet [7] and therefore may potentially show differences in relation to other factors as well, such as feed efficiency and breed. Thus, there is a need to investigate whether the microbiomes in both the rumen and in the feces are equally affected by different factors. The hindgut microbiota is also important for animal health and represents important sources of environmental contamination from feces [8].

Methane is predominately produced by methanogenic archaea residing in the rumen, from the products of rumen fermentation. Bacteria are the most numerous microbes fermenting feedstuffs in the rumen, thus both methanogen and bacterial populations are of interest when examining the influence of factors that might alter methane production. Dietary effects on the rumen microbiome and on methane emission traits are well established, but reports of individual cow effects [9], breed effects, and particularly the effects of interactions between diet and individual animals have been scarcely reported [10]. Residual feed intake (RFI) is the difference between the actual feed intake and the calculated expected feed intake. This can be taken as a measure of efficiency, with more efficient animals eating less than their calculated needs. Animals with a low RFI (high efficiency) have been shown to produce the same amount of methane per day (g of CH4/d) as high-RFI animals on the same diet, but have a higher methane yield (g of CH4/kg of dry matter intake (DMI)) due to the low-RFI cows having lower DMI [11]. Increased efficiency is also linked to lower methane emissions per kg of milk [12]. This led us to speculate that if an animal, already very feed efficient, would gain any further methane reduction from diet modifications. To elucidate this, it is important to examine the interaction of dietary intervention aiming at reducing enteric methane, in combination with cattle breed and the RFI status of the cows as a measure of feed efficiency. We hypothesize that the microbiomes in the rumen fluid and the feces would respond differently to dietary manipulation aimed at reducing methane, and also that the responses would be affected by breed and RFI status.

The aims of the present study were therefore to investigate the microbiome of rumen content and fecal samples from two breeds of dairy cows (Holstein and Jersey), differing in RFI status and fed diets differing in the concentrate-to-forage ratio, and to test the validity of using a fecal sample (easy to obtain) as a proxy for microbial populations/activities in the rumen (hard to obtain).

## 2. Materials and Methods

The experiment was conducted in accordance with the Danish Ministry of Justice, Law No. 726 (9 September 1993). Details on the animal trial have previously been reported by Olijhoek et al. [10]. In brief, 10 Danish Holstein and 10 Danish Jersey dairy cows were fed 2 diets, differing in concentrate proportion (high-concentrate and low-concentrate) in a crossover design of 3 periods with a staggered approach. Animals were housed in tie stalls in the same barn. Prior to the experiment, 20 cows were selected from a herd of 200 cows as either high- or low-efficient animals based on defining RFI using herd data. Details of the RFI calculations have been previously reported by Olijhoek et al. [10]. Diets were composed of grass/clover silage, barley, rapeseed cake, dehulled soybean meal, urea, and mineral and vitamin premixes (Table S1). The low-concentrate (LC) and high-concentrate (HC) diets had a forage-to-concentrate ratio (DM based) of 68:32 and 39:61, respectively. Animals were adapted to the diets for 14 to 26 days in period 1 and for 14 days in periods 2 and 3. Feed samples were collected for calculation of total tract digestibility of nutrients on day 12 and 13 of each period. Methane production was measured using open-circuit respiration chambers, as described by Hellwing et al. [13] on day 15 to 17 of period 1 and day 15 and 16 of periods 2 and 3. Rumen content (15 to 40 mL of both liquid and small particles up to 10 mm) was collected orally using an esophageal probe (FLORA rumen scoop, [14]) at 8 am before new feed was offered on the last day of each period (day 18 of period 1 and day 17 of period 2 and 3) and by an experienced technician. Feces (350 g and subsampled into a 50 mL tube) were collected at the same time as rumen liquid and collected during voluntary defecation or by stimulation. Rumen liquid and fecal samples were stored at −80 °C until DNA extraction. The analysis of VFA in rumen liquid; the DM, NDF, and indigestible neutral detergent fiber (iNDF) in feed and feces; and the calculation of total tract digestibility of NDF using iNDF as internal marker are described and reported in Olijhoek et al. [10].

DNA was extracted from rumen and fecal samples using a Nucleospin Soil DNA extraction kit (Machery-Nagel, Düren, Germany) as per the manufacturer’s instructions, with the following modifications; 700 µL of lysis buffer SL2 was added to 250 µL of resuspended rumen content (both liquid and particles) or 250 mg of feces. Samples were disrupted in a FastPrep-24™ benchtop homogenizer for 2 × 20 s. DNA was eluted in a final volume of 60 µL of elution buffer. The concentration and quality of extracted DNA was measured using Nanodrop ND-1000 spectrophotometer (Thermo Fisher Scientific, Wilmington, DE, USA). PCR amplicon libraries targeting the methyl coenzyme-M reductase (*mcrA*) gene were used to profile the methanogen community from the rumen samples by Terminal-Restriction Fragment Length Polymorphism (T-RFLP) using a method previously described in Zhu et al. [15]. Bacterial communities were assessed with universal primers (Bac341F and Bac805R) [16] covering the V3–V4 regions of the 16S rRNA gene. Amplicon libraries were prepared as described by the Illumina protocol [17] with the following modifications: an additional PCR of 20 cycles to amplify the specific target prior to the incorporation of the Illumina overhang adapters (10 cycles) was performed in duplicate. Duplicate samples were pooled and purified with AMPure XP beads, as described in the protocol. Amplicon libraries were then sequenced on the Illumina MiSeq (Illumina, San Diego, CA, USA) using paired 300 bp reads.

Raw sequence reads were deposited in the NCBI short-read archive (SRA) database under BioProject: PRJNA525989. Bacterial sequence reads underwent quality control, processing, and were clustered into operational taxonomic units (OTU) using the LotuS pipeline [18] with the following options. The sequence truncation length and minimum sequence length after removal of barcodes was 230 bp. The minimum average sequence quality score was 27, the maximum number of ambiguous bases was 0, and maximum homeonucleotide run was set to 8. Sequences were allowed a maximum accumulated error of 0.75. Within the LotuS pipeline, the reads were dereplicated and sequences with a minimum of 2 replicates were retained for OTU clustering. Sequence pairs were merged with FLASH [19] and were clustered into OTUs based on their sequence similarity (97%) with UPARSE [20], and chimeric sequences removed with UCHIME reference-based chimera detection [21]. A representative sequence from each OTU was aligned with ClustalO [22] and a phylogenetic tree built with FastTree2 [23]. Representative sequences, the OTU table, and phylogenetic trees were transferred to QIIME 2 (version 2017.10.0) [24,25], where further analyses were performed. Taxonomy was assigned to each OTU using the RDP classifier with a confidence of 0.8 [26] using greengenes (gg_13_8_otus) as the reference database. Operational taxonomic units that contained fewer than 10 sequences were filtered from the OTU table. Also, 4 rumen samples and 2 fecal samples that had less than 10,000 sequences were filtered from the OTU table. Beta diversity was examined using UniFrac Distance matrices where samples were rarified to 16,500 sequences and visualized with Principal Coordinate Analysis plots (PCoA). The T-RFLP profiles were tested with a non-parametric multiple ANOVA on the Bray–Curtis distances using the ADONIS function in R, (R version 3.2.3, https://www.r-project.org/), allowing for 1000 permutations. Results were considered significant at *p* < 0.05. A principal component analysis was performed in STAMP, version 2.0.9 [27]. Statistical tests on the amplicon sequence data were implemented in QIIME 2. The difference between categorical groups (diet, animal breed, and RFI group) was tested on weighted and unweighted UniFrac distance matrices using PERMANOVA with 999 permutations. To determine significant correlation with the continuous groups, we used mantel tests between a distance matrix created from rumen acetate-to-propionate (A:P) ratio, methane yield (L/kg of DMI), RFI value, or NDF digestibility in total tract, and either the weighted or unweighted UniFrac distance matrix.

## 3. Results

### 3.1. Rumen Methanogen Community

**Figure 1:**
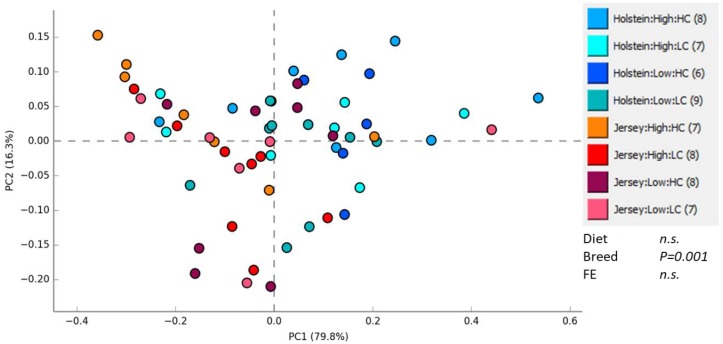
Principal component analysis (PCA) of the methanogen community in the rumen grouped by breed (Holstein or Jersey), residual feed intake (RFI) group (High or Low), and diet (high concentrate: HC; and low concentrate: LC) combinations. Methanogen communities were characterized by T-RFLP analysis on the mcrA gene. The effects of experimental factors were tested using a non-parametric multiple ANOVA on the Bray–Curtis distances.

Results on methane production, rumen fermentation, and nutrient digestibility are reported and discussed in Olijhoek et al. [10]. The rumen methanogen community was profiled by T-RFLP on the methanogen-specific mcrA gene. Principal component analysis (PCA) was performed to visualize the relationship between methanogen communities in the eight treatment groups (breed, RFI group and diet combinations) and is shown in Figure 1. There was a significant difference in the methanogen community related to breed, but no effect of diet or RFI status was detected.

### 3.2. Bacterial Community in the Rumen and Feces

**Figure 2:**
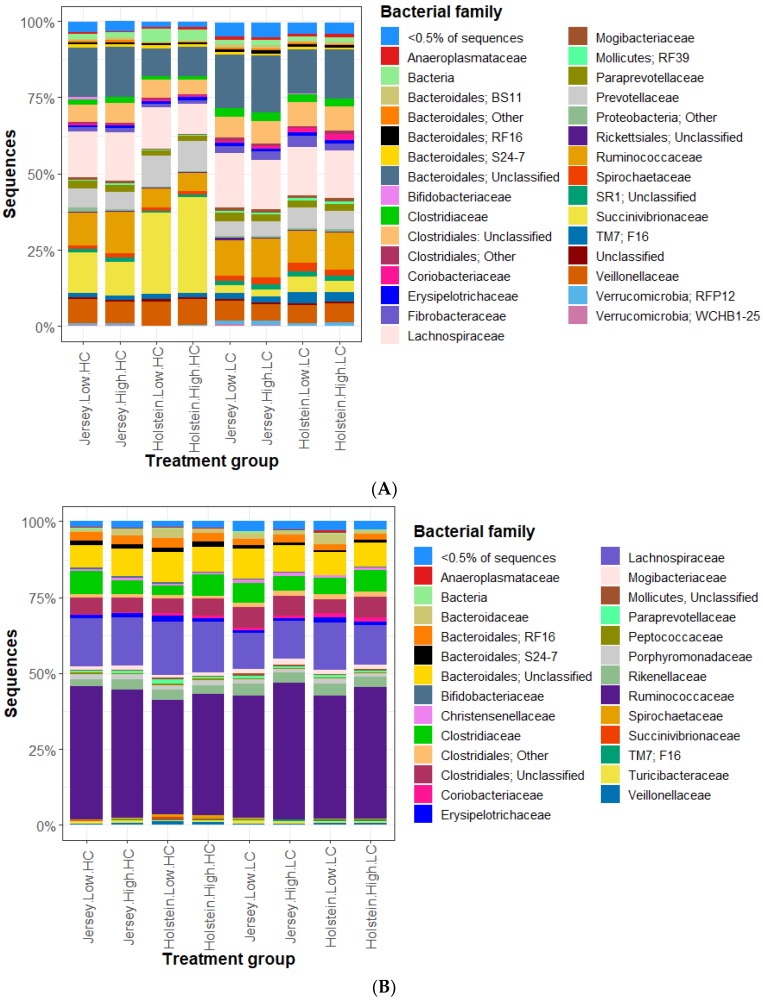
Histogram on relative abundance of bacterial community members in rumen (**A**) and feces (**B**) grouped by breed (Holstein or Jersey), residual feed intake (RFI) group (high or low), and diet (high concentrate: HC; and low concentrate: LC) combinations. Relative abundances are presented at the family or lowest defined level. Genera marked with ’unclassified’ matched to an unclassified sequence, whereas ‘other’ could not be assigned to a group, because there was no close match in the greengenes database.

The bacterial community was profiled by sequencing the V3–V4 region of the 16S rRNA gene. A total of 56 rumen and 58 fecal samples had more than 10,000 good quality sequences each (mean 22,807 sequences for rumen and 29,189 sequences for feces). Sequences were grouped (97% similarity) into 4079 and 2866 OTUs for rumen and fecal samples, respectively. The relative abundance of bacterial families for the eight treatment groups in rumen content and feces are shown in Figure 2. The seven most abundant families present in the rumen samples are: Lachnospiraceae (15% of sequences), unnamed family in Bacteroidales (14.7%), Succinivibrionaceae (12.0%), Ruminococcaceae (10.4%), Veillonellaceae (6.9%), Prevotellaceae (6.7%) and unnamed family in Clostridiales (6.5%). In contrast to this, the most abundant families present in the fecal samples are: Ruminococcaceae (41.5%), Lachnospiraceae (14.9%), unnamed family in Bacteroidales (8.9%), Clostridiaceae (5.8%), unnamed family in Clostridiales (5.6%), Rikenellaceae (3.4%) and Bacteroidales RF16 (2.5%). The rumen bacterial communities exhibited larger differences in relative abundances in relation to treatment groups than the fecal bacterial communities did.

**Figure 3:**
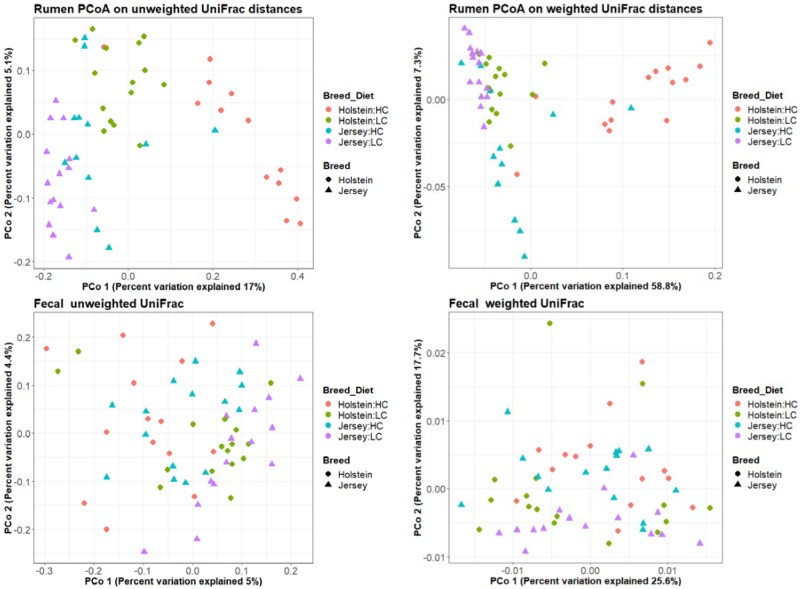
PCoA analysis on UniFrac distances of the microbial communities in rumen (top) and feces (bottom) per breed (Holstein and Jersey) and diet (high-concentrate: HC; and low-concentrate: LC). Left panels are weighted and right panels are unweighted UniFrac distances. Values in parenthesis give the amount of variation explained by each coordinate.

Bacterial community differences between breed and diets are shown with principal coordinate (PCoA) plots in Figure 3 and Table 1 as weighted and unweighted UniFrac distances. Differences caused by breed or diet were obvious in PCoA plots of rumen samples but less clear in fecal communities, although they were statically significant in both except for the effect of breed on the weighted UniFrac in feces (Table 1). A community shift from the transition between high- and low-concentrate diets can be seen as greater within Holstein cows than Jersey cows in both UniFrac measures. The alpha diversity measures (Table S2) show a similar story, where diet was the strongest influence on community differences. Diet caused significant differences in the evenness, observed OTUs, Faith PD and Shannon measures in the rumen and all, except Faith PD, in feces. Differences between breeds were also seen in the evenness measure but only in the rumen samples.

The correlation of rumen or fecal bacterial communities with the continuous traits of NDF digestibility, the acetate-to-propionate (A:P) ratio, methane yield in L/kg of DMI and actual RFI values are shown in Table 2 (values for these traits are reported in [10]). There are stronger correlations between the rumen community and NDF digestibility, A:P ratio and methane than in the fecal bacterial community. Correlations with RFI values were weak and not significant in rumen or feces.

## 4. Discussion

Residual feed intake groups did not differ in methane yield (discussed in Olijhoek et al. [10]), so it is not surprising that RFI status had no effect overall on the methanogen community. This finding is supported by a study in cattle by Zhou et al. [28], who also found RFI status had no effect on the overall methanogen population; likewise, Carberry et al. [29] reported no difference in the abundance of methanogens between RFI phenotypes. Nevertheless, the structure of the methanogen community, detected by examining DNA, does not always indicate differences in methane emission as this may be determined at the gene expression level rather than the community structure level [30]. In our case, breed but not diet had a significant effect on the methanogen community structure, but both breed and diet had a significant influence on the methane yield expressed as L/kg of DMI. However, only diet had an effect on methane production when expressed per kg of energy-corrected milk (ECM). Our results are in contrast to Carberry et al. [29] and Jeyanathan et al. [31], where diet affected the methanogen populations and Cersossimo et al. [32], where breed had no effect on methanogen populations. However, our results are in agreement with Kumar at al. [33], where diet had no influence on methanogen populations.

The bacterial community plays an important role in methane emissions by producing the substrates for methanogenesis via the conversion of feed to fermentation products. In this study, the overall bacterial communities are dominated by Lachnospiraceae, Bacteroidales and Succinivibrionaceae, where Prevotellaceae is ranked 6th with only 6.7% of the sequences. This seems low as Prevotellaceae is usually the most dominant genus in the rumen [34]. This may reflect real differences in our experimental animals or biases in the sample handling and sequencing methodologies, but similar results were reported from animals in this herd by Zhu et al. [15]. Our samples were taken orally with a stomach probe, a method that allows rumen sampling from an animal that has not been surgically altered, and although this allowed fine feed particles to be collected, large solid digesta was omitted. The inclusion of solid feed particles is important for good representation of the rumen community as the majority of bacteria adhere to the feed particles [35]; however, it has been demonstrated that community composition of the ruminal liquid phase observed from the stomach probe technique is indistinguishable from those collected via rumen cannula [36,37].

Diet is known to have a prominent effect on the bacterial population structure [34,38,39] as this provides the substrates for bacterial growth and therefore determines selective pressure on the community. Diet had a large impact on the bacterial communities, showing a significant effect in both the rumen and the feces with both weighted and unweighted UniFrac measures (Table 1). Diet also strongly affected the alpha diversity measures of both rumen and fecal communities (Table S2). Weighted UniFrac distances take into consideration the number of sequences in each OTU and are suitable for showing changes in taxon abundance. They usually show shifts in the dominant taxa, whereas unweighted UniFrac distances are suitable for showing differences in community membership. The latter usually show the presence or absence of the less dominant taxa as it is unlikely in the gut environment that a dominant group will disappear altogether. Diet-related community differences were more pronounced for Holstein cows than for Jersey cows (Figure 3) demonstrating differences in breed response to the diets.

The bacterial community differed between Holstein and Jersey cows for both weighted and unweighted UniFrac measures in the rumen, but were only significantly different in the unweighted UniFrac measure in feces. This demonstrates that breed-related differences in the bacterial community are evident in the rumen; however, they are less pronounced in the feces, where they may only differ in the less dominant taxa. A previous study by Paz et al. [37], comparing the rumen communities between Holstein and Jersey cows, also reported community differences between breeds. Contrary to this, Bainbridge et al. [40] found that breed contributed to very few differences in the rumen community when comparing Holstein, Jersey and Holstein–Jersey crossbreds on the same diet.

Few studies have looked at both rumen and fecal communities concurrently; however, one study, examining high- vs low-production dairy cattle, observed differences in the ruminal bacteria but not in the feces [41]. In another case, Dill-McFarland et al. [42] found the effect of animal age strongly influenced both the rumen and fecal bacterial communities, but in adult cows only the rumen communities were affected by diet. We found that both animal breed and diet affect the rumen community, where breed was only significant with the unweighted UniFrac in the fecal community. We also found that rumen communities have a much stronger correlation with rumen fermentation parameters (NDF digestibility, A:P ratio and methane production) than fecal communities. Bacterial communities associated with each animal were statistically different for the unweighted UniFrac in both rumen and feces, but only feces showed animal differences in the weighted UniFrac. These results indicate that the animal-specific microbes mostly comprise minor groups in the rumen, but animal-to-animal variation is more pronounced in fecal communities. Animal-specific variation in rumen bacterial communities is dependent on the animals chosen as it is significant in some studies [1] but not in other studies [43]. Therefore, examining the fecal communities may have an advantage when looking at animal differences.

No differences were detected between the two RFI groups in either rumen or fecal bacterial communities (Table 1). In addition, there was no correlation between the actual RFI values and either the rumen or fecal communities (Table 2). Therefore, we cannot confirm our hypothesis that RFI status would correlate with changes in the microbial community. Without any effect of RFI status, we cannot determine whether cows that are more efficient would respond differently to dietary interventions aimed at reducing methane than less efficient cows. This study used animals from an experimental herd, simulating a commercial herd. Therefore, the genetic differences and differences in RFI were small and are comparable to those of the whole Danish Holstein and Jersey populations. The microbiome results reported here are in concordance with the lack of significant differences seen in methane production, rumen fermentation, and milk characteristics in connection with RFI, as reported by Olijhoek et al. [10].

The rumen bacterial community plays a significant role in NDF digestibility, VFA production, and methane emission. Moderate and significant correlations between rumen communities to the total tract NDF digestibility, rumen A:P ratio and methane yield per kg of DMI are evident, with mantel correlation tests (Table 2). This suggests a link between the bacterial community and methane production. In accordance, Kittelmann et al. [44] also found that differences in rumen bacterial communities were linked to methane emissions. In the present study, correlations were weaker in the fecal communities compared to the rumen community. The fecal communities have a significant but weak correlation with NDF digestibility, rumen A:P ratio and methane production with a stronger correlation for the unweighted UniFrac. Overall, these results indicate that differences between taxonomic groups in the feces are smaller compared to those in rumen samples.

## 5. Conclusions

Changes in methanogen communities did not relate to differences in methane production, but the structure of the bacterial community was correlated with methane production. Community differences seen in the rumen are reduced or absent in feces except in the case of animal-to-animal variation, where differences were more pronounced. Therefore, feces samples are not representative of the differences seen in rumen communities and should not be used as proxies for the latter. Changes in the bacterial communities were observed with diet intervention and between breeds but not with differing RFI status. The two cattle breeds had different levels of response to the dietary intervention; therefore, it may be appropriate to tailor methane reduction strategies to each cattle breed individually.

## Figures and Tables

**Table 1:** Effect of breed, animal, diet, and residual feed intake (RFI) group on the beta diversity of bacteria in rumen and feces.

Factor	Rumen ^1^		Fecal ^1^	
	Unweighted UniFrac	Weighted UniFrac	Unweighted UniFrac	Weighted UniFrac
Breed	0.001	0.001	0.001	0.141
Animal	0.002	0.147	0.001	0.001
Diet	0.001	0.001	0.001	0.002
RFI groups	0.846	0.673	0.499	0.398

^1^ Results are *p*-values of a PERMANOVA test with 999 permutations.

**Table 2:** Correlation of bacterial community structure to the animal traits: NDF digestibility in total tract, acetate-to-propionate (A:P) ratio in rumen liquid, methane yield (L/kg dry matter intake (DMI)), and residual feed intake (RFI) values.

Trait	Rumen ^1^				Fecal ^1^			
	Unweighted UniFrac		Weighted UniFrac		Unweighted UniFrac		Weighted UniFrac	
	r	*p*	r	*p*	r	*p*	r	*p*
NDF digestibility	0.384	0.001	0.475	0.001	0.33	0.001	0.188	0.002
A:P ratio	0.577	0.001	0.654	0.001	0.302	0.001	0.127	0.001
Methane ^2^	0.492	0.001	0.556	0.001	0.364	0.001	0.163	0.006
RFI values	0.009	0.842	0.0001	0.99	0.054	0.347	0.008	0.891

^1^ Correlations (r) and significance (*p*) were determined by Mantel tests between the bacterial community structures represented as weighted or unweighted UniFrac distances and animal trait variables. ^2^ L/kg of DMI.

## Supplementary Materials

The following are available online at https://www.mdpi.com/2076-2615/9/8/498/s1, Table S1: Dietary and chemical composition (g/kg DM unless noted) of the low-concentrate (LC) and high-concentrate (HC) diets, Table S2: Effect of breed, animal, diet, and residual feed intake (RFI) group on the alpha diversity of bacteria in rumen and feces.
