# Supplementary material for: Rumen and Fecal Microbial Community Structure of Holstein and Jersey Dairy Cows as Affected by Breed, Diet, and Residual Feed Intake
Source: Animals (Basel). 2019 Jul 29;9(8):498. doi: 10.3390/ani9080498 (PMC6721167; doi:10.3390/ani9080498)
Supplement: Supplementary file 1 [file animals-09-00498-s001.pdf]

Table S1. Dietary and chemical composition (g/kg DM unless noted) of the low-concentrate (LC) and high-concentrate (HC) diets.

**Item**	**LC**	**HC**
F:C ratio [1]	68:32	39:61
Dietary composition		
Grass/clover silage	684	391
Barley	189	377
Rapeseed cake	25.7	51.4
Soybean meal, dehulled	85.7	171
Urea	4.7	2.7
Mineral premix [2]	9.3	5.3
Vitamin premix [3]	2.1	1.2
Chemical composition		
DM (g/kg)	513	620
Ash	72.0	57.3
CP	170	204
Crude fat	31.8	33.6
Starch	105	218
NDF	335	271
iNDF [4]	45.3	41.8
Gross energy (MJ/kg of DM) [5]	18.7	19.2

[1] F:C ratio = forage:concentrate ratio. [2] Vitamin and mineral concentration per kilogram DM of feedstuff: 176 g of chloride, 140 g of magnesium, 120 g of calcium, 120 g of sodium, 1.5 g of potassium, 0.5 g of sulfur, 4500 mg of zinc, 4000 mg of manganese, 1500 mg of copper, 225 mg of iodine, 50 mg of selenium, 25 mg of cobalt, 600,000 IE of vitamin A, 190,000 IE of vitamin D3, 4000 IE of vitamin E. [3] Vitamin and mineral concentration per kilogram DM of feedstuff: 10 mg of selenium, 5,000,000 IE of vitamin A, 200,000 IE of vitamin D, 10,000 IE of vitamin E. [4] iNDF = indigestible NDF. [5] Calculated according to NorFor (Volden and Nielsen, 2011).

Table S2. Effect of breed, animal, diet, and residual feed intake (RFI) group on the alpha diversity of bacteria in rumen and feces.

	**Metric**	**Animal**	**Breed**	**Diet**	**RFI**
Rumen [1]	Evenness	0.659	0.008	<0.001	0.929
	Observed OTUs	0.704	0.058	<0.001	0.709
	Faiths PD	0.251	0.247	0.003	0.444
	Shannon	0.691	0.012	<0.001	0.957
Feces [1]	Evenness	0.107	0.479	0.003	0.243
	Observed OTUs	0.105	0.436	<0.001	0.191
	Faiths PD	0.127	0.895	0.112	0.554
	Shannon	0.086	0.414	<0.001	0.283

[1] Results are P-values of PERMANOVA test with 999 permutations.
